# Supplementary figures and images for: The effect of waning on antibody levels and memory B cell recall following SARS-CoV-2 infection or vaccination
Source: bioRxiv. 2022 Mar 17:2022.03.16.484099. Preprint. [Version 1] doi: 10.1101/2022.03.16.484099 (PMC8936119; doi:10.1101/2022.03.16.484099)

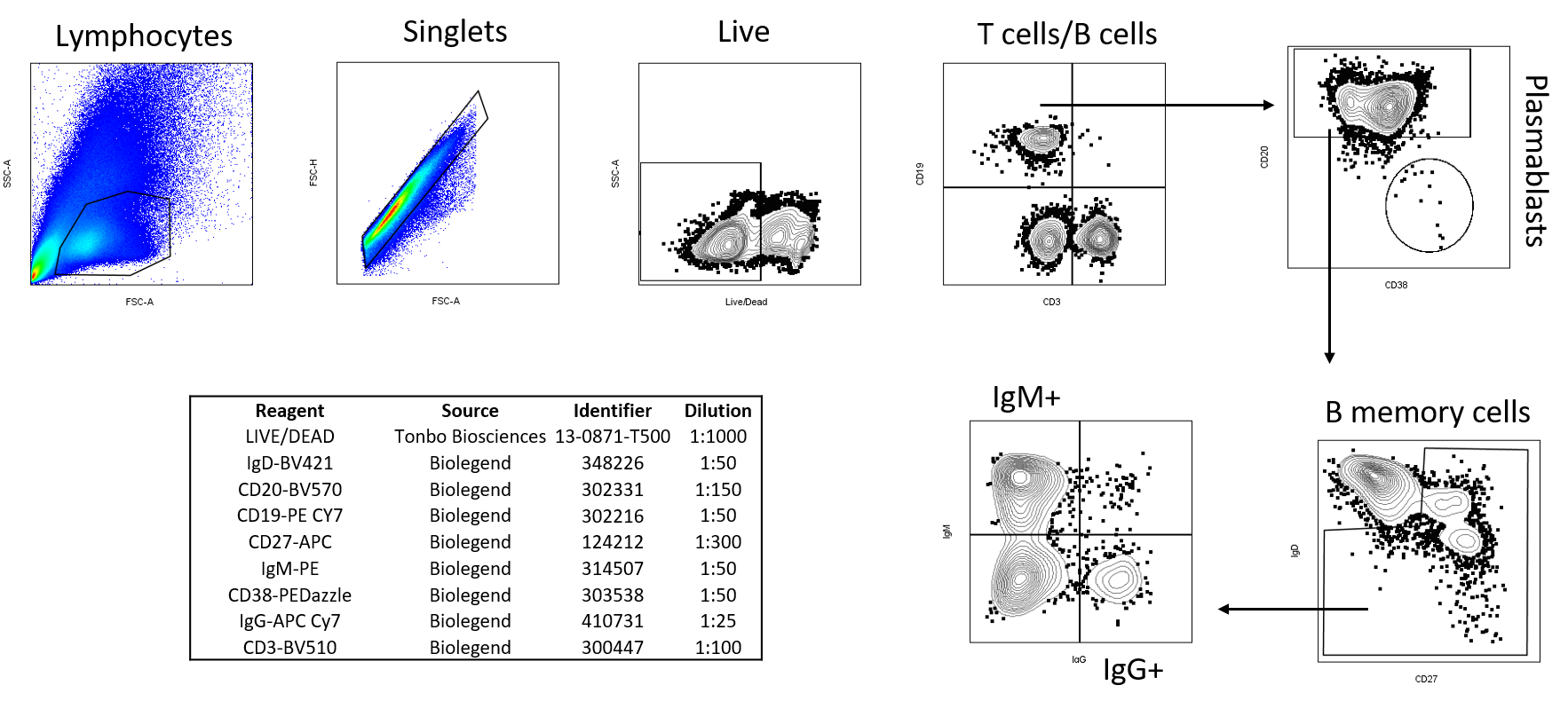

Supplement: Supplement 4 — Figure S1: Memory B cell gating strategy. [file media-4.tif]
